# Effects of acute ingestion of different fats on oxidative stress and inflammation in overweight and obese adults

**DOI:** 10.1186/1475-2891-10-122

**Published:** 2011-11-07

**Authors:** Abigail D Peairs, Janet W Rankin, Yong Woo Lee

**Affiliations:** 1 Department of Human Nutrition, Foods, and Exercise, Virginia Polytechnic Institute and State University, Blacksburg, VA 24061. USA; 2 School of Biomedical Engineering and Science, Virginia Polytechnic Institute and State University, Blacksburg, VA 24061. USA

**Keywords:** meal challenge, postprandial, endothelial activation, obesity, NF-κB

## Abstract

**Background:**

Studies show that obese individuals have prolonged elevations in postprandial lipemia and an exacerbated inflammatory response to high fat meals, which can increase risk for cardiovascular diseases. As epidemiological studies indicate an association between type of fat and circulating inflammatory markers, the purpose of this study was to investigate the acute effect of different fat sources on inflammation and oxidative stress in overweight and obese individuals.

**Methods:**

Eleven overweight and obese subjects consumed three high fat milkshakes rich in monounsaturated fat (MFA), saturated fat (SFA), or long-chain omega 3 polyunsaturated fat (O3FA) in random order. Blood samples collected at baseline, 1, 2, 4, and 6 hours postprandial were analyzed for markers of inflammation (soluble intercellular adhesion molecule-1 (ICAM-1), vascular cell adhesion molecule-1 (VCAM-1), tumor necrosis factor- α (TNF-α), and C-reactive protein (CRP)), oxidative stress (8-epi-prostaglandin-F2α (8-epi) and nuclear factor-κB (NF-κB)), and metabolic factors (glucose, insulin, non-esterified free fatty acids, and triglycerides (TG)).

**Results:**

O3FA enhanced NF-kB activation compared to SFA, but did not increase any inflammatory factors measured. Conversely, SFA led to higher ICAM-1 levels than MFA (p = 0.051), while MFA increased TG more than SFA (p < 0.05). CRP increased while TNF-α and 8-epi decreased with no difference between treatments.

**Conclusions:**

While most of the inflammatory factors measured had modest or no change following the meal, ICAM-1 and NF-κB responded differently by meal type. These results are provocative and suggest that type of fat in meals may differentially influence postprandial inflammation and endothelial activation.

## Background

Cardiovascular disease and type 2 diabetes are associated with obesity and are also linked to inflammation and oxidative stress [1]. Weight loss is effective in reducing these conditions [2], however, as only 1 in 5 overweight people successfully maintain weight loss [3], alternative dietary strategies to improve health without weight loss are desirable.

Most of each day is spent in the postprandial state. Increases in blood glucose and/or triglycerides following a meal stimulate oxidative stress, impair endothelial function, and cause a rise in circulating inflammatory factors [4]. Research suggests that the negative postprandial responses are exaggerated in obesity and diabetes [4-6]. The repeated acute stresses induced by food ingestion (particularly high fat and/or high calorie meals) may contribute to acute cardiac events and/or stimulate further development of atherosclerosis [7,8]. Although most clinical evaluation of cardiovascular disease risk is based on fasted blood values, muting of the excursions in atherogenic factors during the postprandial period could have important health consequences [9].

Previous research has shown that chronic ingestion of specific fats, such as saturated fats, increase cardiovascular disease risk while other fats, including long chained omega 3 fats (n-3FA), reduce risk [10,11]. The differential effect of these fats on chronic disease risk has been hypothesized to be mediated by unique effects on blood lipids, hemostasis, endothelial function, or inflammation [12]. For example, Tholstrup et al [13] observed different postprandial lipemic responses when they evaluated six meals differing in type of fat. In general, the longer and more saturated fats caused delayed and lower increase in plasma fats. One laboratory recently reported that acute monounsaturated fat ingestion caused more impairment of flow mediated vasodilation than a high saturated fat meal [14] and another group showed that inclusion of a fish oil supplement helped to preserve endothelial function following a high fat meal [15]. Another group also reported improved vascular reactivity following a fish oil meal compared to a mixed fat meal, with a potential role for reduced oxidative stress [16].

Oxidative stress is hypothesized to be a significant mediator of impairment in postprandial endothelial function as well as a stimulator of the inflammatory response following a high fat meal [17,18]. For example, Nappo et al [19] and Carroll et al [20] reported that ingestion of antioxidant vitamins blunted or eliminated the postprandial rise in inflammatory factors in type 2 diabetics. Since specific types of FA can differentially affect oxidative stress due to differences in chemical susceptibility to oxidation, it is of interest to determine whether acute ingestion of fats differing in number of double bonds influences the postprandial inflammatory response. Bellido et al [21] observed an increase in activation of a redox-sensitive transcriptional factor, NF-kB, in peripheral blood mononuclear cells following a meal high in butter or walnuts but not olive oil. This is intriguing and requires additional study to determine specific fatty acid effects, and information on the effects of meals specifically enriched with n-3FA. The acute effects of n-3FA on oxidative stress and inflammation have not been extensively studied, especially in overweight individuals who may be more susceptible to inflammatory stimuli.

In summary, diets high in n-3FA are typically associated with lower systemic markers of inflammation in many epidemiological studies [11], but there has been limited research with conflicting results concerning the acute effect of n-3FA ingestion on inflammatory response after a meal. As most of the day is spent in the postprandial state, and obese individuals experience greater oxidative stress and inflammatory responses to high fat meals compared to lean individuals, it is of interest to determine whether meal composition affects the postprandial inflammatory response. The objective of this study was therefore to clarify the role of different sources of fat in a high fat meal on inflammation and oxidative stress in overweight and obese adults.

## Methods

### Subject Selection

Eleven overweight and obese (BMI > 27 kg/m^2^), non-smoking, sedentary, weight stable adult subjects were recruited for this study. All subjects signed informed consent that had been approved by the Virginia Tech Institutional Review Board for Human Subjects after receiving full explanation of the procedures involved with this study. Subjects were excluded if they had any past or present cardiovascular disease, diagnosed diabetes or inflammatory condition, or were taking medications known to affect inflammation. Any use of dietary supplementation (i.e. antioxidants, vitamins/minerals, fish oil) ceased at least 2 weeks prior to starting the study.

### Study design

Each subject completed three meal trials in a randomized, cross-over design with at least one week between trials first thing in the morning. Subjects were instructed to follow the same pattern of eating for the 3 days prior to each test day. Blood was collected from overnight fasted subjects prior to each test meal (time 0) as well as 1, 2, 4, and 6 hours after meal consumption via repeated venipuncture. Subjects remained in the laboratory and were not allowed to consume any additional foods or beverages except water in the postprandial period.

### Meal composition

All meals were high energy, high fat milkshakes, similar in energy and macronutrient composition, with the exception of the source and type of fat. The meals were high in mono-unsaturated fat, saturated fat, or contained a substantial dose of n-3FA. The fat source was blended with 1% milk, strawberry flavored syrup, low fat frozen yogurt, and non-fat dry milk powder. The fat sources in the meals were the following: refined olive oil (MFA), refined palm oil (SFA), and refined olive oil plus 4 g of n-3FA from 8 g fish oil supplement pills (O3FA, Vitamin World Super Omega-3 Fish Oil containing 300 mg EPA, 200 mg DHA per 1 g). The milkshakes contained approximately 12.8 kilocalories per kilogram body weight with 59% fat, 30% carbohydrate, and 11% protein. The meals averaged 1267 calories with the O3FA adding 36 calories. Two subjects were unable to finish their entire milkshake the first day, therefore, the remaining two milkshakes for those subjects were adjusted to match the amount consumed the first day (these were still substantial meal challenges providing 9-10 kcal/kg).

### Blood collection and processing

Blood was collected into vacutainers containing anticoagulant at 0, 1, 2, 4, and 6 hours postprandial and immediately placed on ice until processing. Blood was centrifuged (1000 × g), to obtain plasma for analysis of inflammatory, oxidative stress, and metabolic parameters. Plasma was aliquoted into separate cryovials for each measure and immediately stored at -80°C. For isolation of peripheral blood mononuclear cells (PBMC), heparinized blood was mixed 1:1 with cold PBS and layered over lymphocyte separation media (LSM) (Mediatech) for density gradient separation of PBMCs. PBMCs were harvested, rinsed twice, and subjected to the nuclear extraction protocol for measurement of nuclear factor - κB (NF-κB). Cells were kept on ice throughout the procedure. Nuclear extracts from PBMCs were prepared according to the method of Andrews and Faller [22] with minor modifications. PBMCs were lysed in 400 μL buffer A (10 mM HEPES-KOH pH 7.9, 1.5 mM MgCl2, 10 mM KCl, 0.5 mM DTT, 0.2 mM PMSF), followed by centrifugation to obtain the nuclear pellet which was incubated in 20-40 μL (depending on the pellet size) of buffer C (20 mM HEPES K-OH pH 7.9, 25% glycerol, 420 mM NaCl, 1.5 mM MgCl2, 0.2 mM EDTA, 0.5 mM DTT, 0.2 mM PMSF) on ice. After centrifugation, the supernatant was collected, aliquoted, and immediately frozen at -80°C.

### Inflammatory marker analysis

Plasma was analyzed in duplicate for soluble intercellular adhesion molecule-1 (ICAM-1), soluble vascular cell adhesion molecule-1 (VCAM-1), tumor necrosis factor- α (TNF-α) (all R&D systems Minneapolis, MN), and C-reactive protein (CRP) (United Biotech, Mountain View, CA) via enzyme linked immunosorbent assays (ELISA).

### Oxidative stress marker analysis

Both markers of oxidative stress, nuclear factor- κB (NF-κB) and 8-epi-prostaglandin-F2α (8-epi), were analyzed for 4 h postprandially as per previously reported rapid response within 1-4 h [23,24]. 8-epi was determined using gas chromatography mass spectrometry (GC/MS) by the Morrow laboratory according to the methodology of Milne and Morrow [25]. Briefly, free F2- isoprostanes were extracted from 1 ml of plasma. Deuterated [^2^H4]-8-iso- PGF2 α internal standard was added, vortexed, applied to a C18 Sep-Pak column, and extracted. F2-isoprostanes were converted into pentafluorobenzyl esters, subjected to thin-layer chromatography, extracted from the silica gel with ethyl acetate, converted into trimethylsilyl ether derivatives and analyzed by negative ion chemical ionization GC/MS using an Agilent 5973 mass spectrometer with a computer interface.

An electrophoretic mobility shift assay (EMSA) procedure was performed to measure NF-κB activation with as previously described [26]. Binding reactions were conducted in a 20 μl volume containing 2-4 μg (4 μL) of nuclear protein extracts, 10 mM Tris-Cl, pH 7.5, 50 mM NaCl, 1 mM EDTA, 0.1 mM dithiothreitol, 10% glycerol, 2 μg of poly[dI-dC] (nonspecific competitor) and 40, 000 cpm of ^32^P-labeled specific oligonucleotide probe. Double-stranded oligonucleotides with the consensus sequence of the binding site for transcription factor NF-κB(5'-AGTTGAGGGGACTTTCCCAGG-3', (Promega, Madison, WI)) were labeled with [γ-^32^P]-ATP (Amersham Pharmacia Biotech, Piscataway, NJ) using T4 polynucleotide kinase. Resultant protein-DNA complexes were resolved on native 5% polyacrylamide gels using 0.25 × TBE buffer (50 mM Tris-Cl, 45 mM boric acid, 0.5 mM EDTA, pH 8.4). Competition studies were performed by the addition of a molar excess of unlabeled oligonucleotide to the binding reaction. Gels were dried and exposed to x-ray film. Band intensity corresponding to specific NF-κB-DNA binding was determined using Syngene GeneTools software (Imgen, Alexandria VA). The relative intensity units were calculated in relation to the basal value for each subject for each trial.

### Metabolic variables

Plasma glucose (Stanbio, Boerne, TX), non-esterified fatty acids (NEFA), and triacylglycerol (TG) were measured spectrophotometrically with the latter two analyzed using a technique adapted for microplate (Wako, Richmond, VA). Insulin was measured by ELISA (Mercodia, Uppsala, Sweden).

### Statistics

Any subject with more than one missing data point in more than one trial for more than one measure was excluded from the overall analysis. This resulted in one subject being excluded, therefore data are presented for n = 10. Data were analyzed for the effects of treatment, time, and the treatment by time interaction by two factor RM-ANOVA using a mixed linear model and the baseline values as covariates. Treatment and time were fixed effects and subject was a random effect. Non-normally distributed data were log transformed prior to analysis (CRP, 8-epi, TG). For clarity, the measured data are presented in figures. The area under the curve (AUC) for each dependent measure was calculated using the linear trapezoidal method and analyzed for difference by treatment via a one factor ANOVA using a mixed linear model with the baseline values as covariates. Post hoc analyses were conducted only when a significant effect was detected with ANOVA to determine which treatments or time points were different. Data are presented as mean ± standard error of the mean (SEM), a p-value ≤ 0.05 was considered significant, and all analyses were carried out using SPSS version 15.0 (SPSS, Chicago IL).

## Results

### Fasting measurements

There were no reported differences in dietary intake or physical activity level between meal trials. Initial subject characteristics and average fasting levels (of the 3 baseline values) for inflammatory, oxidative stress, and metabolic variables are located in Table 1. Data are also presented by gender, however, as gender was not included in the statistical analysis due to a modest subject number, it is presented for exploratory purposes only. Waist circumference was positively correlated with both average fasted plasma glucose (r = 0.713, p = 0.014) and TG (r = 0.645, p = 0.032) levels, while body mass index was positively correlated with several markers of inflammation including plasma levels of TNF-α (r = 0.661, p = 0.027), CRP (r = 0.857, p = 0.007), and ICAM-1 (r = 0.778, p = 0.008). Fasted glucose and TG were strongly correlated (r = 0.930, p < 0.001), while insulin tended to correlate with ICAM-1 (r = 0.652, p = 0.052). Fasted CRP was positively correlated with both ICAM-1 (r = 0.838, p = 0.002) and the oxidative stress marker 8-epi (r = 0.734, p = 0.016). Other combinations of fasting measures did not exhibit significant correlations (p > 0.05).

**Table 1:** Subject characteristics and average fasting values for dependent measures

Measure	All subjects		Male Subjects	Female subjects
	**Mean ± SEM**	**Range**	**Mean ± SEM**	**Mean ± SEM**

N	10	-	4	6

Age (years)	31.3 ± 3.3	21-47	38.5 ± 4.2	29.5 ± 4.4

Weight (kg)	98.6 ± 5.7	71-124	107.7 ± 6.4	92.6 ± 8.0

Waist (cm)	99.5 ± 4.8	78-122	108.8 ± 5.4	93.3 ± 6.2

Body Mass Index (kg/m^2^)	34.6 ± 2.0	27-45	33.0 ± 1.5	35.6 ± 3.2

C-reactive protein (mg/L)	3.5 ± 1.4	0.3-10.0	2.2 ± 1.1	4.7 ± 1.5

Tumor necrosis factor-α (pg/mL)	1.30 ± 0.12	0.85-1.64	1.26 ± 0.21	1.32 ± 0.15

Intercellular adhesion molecule-1 (ng/mL)	210 ± 11	163-269	194 ± 14	220 ± 15

Vascular cell adhesion molecule-1 (ng/mL)	677 ± 42	447-924	658 ± 52	690 ± 65

8-epi-prostaglandin-F2α (pg/mL)	0.08 ± 0.01	0.05-0.14	0.06 ± 0.01	0.10 ± 0.01

Glucose (mmol/L)	6.0 ± 0.3	4.8-8.5	6.8 ± 0.6	5.5 ± 0.2

Insulin (mU/L)	11.0 ± 2.1	4.8-26.4	10.0 ± 2.2	11.6 ± 3.3

Triglycerides (mmol/L)	1.58 ± 0.47	0.63-5.50	2.52 ± 1.08	0.95 ± 0.13

Non-esterified fatty acids (mmol/L)	0.33 ± 0.04	0.12-0.49	0.34 ± 0.08	0.32 ± 0.04

### Postprandial Responses

#### Inflammatory Markers

**Figure 1:**
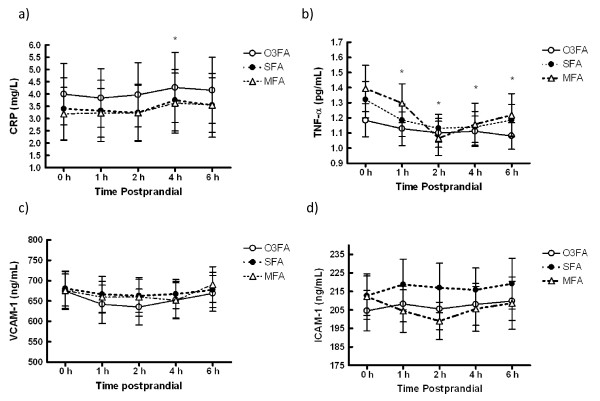
**Inflammatory mediator response to three high fat meals differing in fat type**. Plasma a) C-reactive protein (CRP) increased over time (p < 0.05), b) tumor necrosis factor-α (TNF-α) decreased over time (p < 0.001), c) Soluble vascular cell adhesion molecule-1 (VCAM-1) tended to decrease over time (p < 0.085), and d) Soluble intercellular adhesion molecule-1 (ICAM-1) but remained lower for MFA (p = 0.031) than both O3FA and SFA, and the area under the curve was higher for SFA than MFA (p = 0.051) as assessed by ELISA. Data are presented as Mean ± SEM. O3FA = omega 3 fatty acid enriched meal, SFA = high saturated fat meal, and MFA = high monounsaturated fat meal. * indicates time point difference from baseline (p < 0.05).

Plasma CRP increased slightly over time (p = 0.045) with no difference between meals (Figure 1a), while TNF-α decreased and VCAM-1 tended to decrease following the meals (p < 0.01 and p = 0.085 respectively) with no difference between treatments (Figure 1b and Figure 1c). Plasma ICAM-1 did not change significantly following the meals examined together, but remained lowest for the MFA treatment (p = 0.031), and the ICAM-1 AUC was greater for SFA than MFA (p = 0.051) (Figure 1d).

#### Oxidative Stress Markers

**Figure 2:**
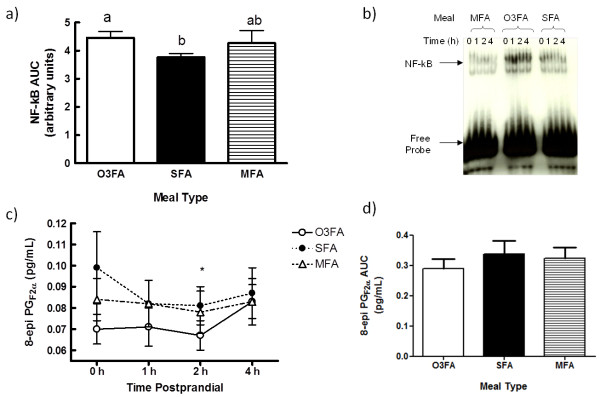
**Oxidative stress marker response to three high fat meals differing in fat type**. Over 4 h, a) the area under the curve for peripheral blood mononuclear cell nuclear factor-κB (NF-κB) activation was higher for O3FA than SFA (p < 0.05) as determined by densitometric data analysis following EMSA analysis and b) shown in a representative EMSA image of NF-κB activation. Also, c) plasma 8-epi prostaglandin_F2α _(8-epi) analyzed by GC/MS decreased over time with no difference between groups (p < 0.02) and d) no difference in area under the curve. Data are presented as Mean ± SEM. O3FA = omega 3 fatty acid enriched meal, SFA = high saturated fat meal, and MFA = high monounsaturated fat meal. * indicates time point difference from baseline. ^a, b ^Difference in superscript denotes difference between meals as the area under the curve for O3FA > SFA (p<0.05)..

Although there was no significant combined-meal postprandial effect on NF-κB, it was higher throughout the 4 h postprandial period for O3FA than SFA, resulting in a greater AUC (p = 0.046, Figures 2a and 2b). 8-epi decreased during the postprandial period (p = 0.019) with no difference between treatments (Figure 2 c and 2d).

#### Metabolic Variables

As expected, there were significant effects of meal ingestion on plasma insulin (p < 0.001), glucose (p = 0.019), NEFA (p < 0.001), and TG (p < 0.001) (Table 2). Insulin and glucose increased 1 h postprandial and then returned to baseline levels. NEFA had the opposite trend, decreasing initially and then rebounding to baseline, while TG increased steadily over time. Type of fat in the meal did not influence the plasma glucose or NEFA response. However, the insulin AUC tended to be lower for SFA than O3FA (p = 0.076) while TG were lower for SFA than MFA (p = 0.019).

**Table 2:** Postprandial (PP) metabolic measures (mean ± SEM) following three meals differing in fat type (n = 10)

			Time point			Area under
Measure	Baseline	1 h PP	2 h PP	4 h PP	6 h PP	the curve
Glucose (mmol/L)*^a^						

O3FA	5.9 ± 0.3	6.7 ± 0.5	6.1 ± 0.5	5.7 ± 0.3	6.2 ± 0.5	36.4 ± 2.0

SFA	6.2 ± 0.5	6.2 ± 0.7	6.4 ± 0.6	5.9 ± 0.5	5.9 ± 0.4	36.5 ± 3.0

MFA	5.8 ± 0.2	6.2 ± 0.5	5.4 ± 0.3	5.7 ± 0.2	5.9 ± 0.2	34.4 ± 1.3

Insulin (mU/L)*^b^						

O3FA	12.6 ± 3.8	54.8 ± 14.8	43.4 ± 10.9	16.4 ± 3.2	11.5 ± 1.9	171 ± 36

SFA	8.9 ± 1.2	46.9 ± 11.9	29.0 ± 6.2	10.7 ± 1.0	9.8 ± 2.3	126 ± 20

MFA	11.5 ± 2.2	40.2 ± 10.5	33.1 ± 8.3	18.6 ± 4.4	14.7 ± 4.3	148 ± 34

Triglycerides (mmol/L) †* ^c^						

O3FA	1.49 ± 0.45	1.79 ± 0.45	1.61 ± 0.40	1.92 ± 0.38	2.28 ± 0.59	11.08 ± 2.52

SFA	1.42 ± 0.38	1.34 ± 0.27	1.24 ± 0.26	1.63 ± 0.30	1.65 ± 0.30	8.83 ± 1.69

MFA	1.82 ± 0.63	2.27 ± 0.66	2.25 ± 0.58	2.41 ± 0.59	2.79 ± 0.74	13.97 ± 3.68

Non-esterified fatty acids (mmol/L)*^d^						

O3FA	0.33 ± 0.05	0.17 ± 0.04	0.10 ± 0.02	0.20 ± 0.02	0.39 ± 0.05	1.27 ± 0.15

SFA	0.37 ± 0.06	0.10 ± 0.02	0.08 ± 0.02	0.21 ± 0.03	0.39 ± 0.05	1.22 ± 0.15

MFA	0.29 ± 0.05	0.15 ± 0.06	0.14 ± 0.05	0.20 ± 0.02	0.32 ± 0.04	1.21 ± 0.20

O3FA = omega 3 fatty acid enriched meal, SFA = high saturated fat meal, and MFA = high monounsaturated fat meal

† Effect of treatment p < 0.05, MFA > SFA

* Effect of time p < 0.05

^a ^1 h PP > all other time points

^b ^1 h PP > all time points, 2 h PP > all except 1 h

^c ^baseline < all PP time points

^d ^baseline > all time points except 6 h PP

## Discussion

The key findings of this study were that the type of fat in a high fat meal differentially influenced levels of the redox sensitive transcription factor NF-κB and the soluble adhesion molecule ICAM-1 in overweight and obese individuals. The observation in our study that a meal containing a substantial dose of n-3FA (DHA +EPA) led to higher levels of NF-κB than a meal high in saturated fat is intriguing since generally epidemiological and *in vitro *studies support an anti-inflammatory effect of these fats [12]. For example, pre-exposure to n-3FA reduced pro-inflammatory responses in vascular endothelial cells [27]. However, other *in vitro *studies have reported that exposure to EPA increases or prolongs cell-stimulated NF-κB activation [28,29] It is not necessarily surprising that the acute responses to n-3FA are different than to chronic ingestion because repeated supplementation allows for the incorporation of n-3FA into cell membranes. n-3FA partially replace arachidonic acid and result in the production of the less prothrombic and inflammatory n-3FA-derived metabolites than those produced from arachidonic acid [11,30]. Cell exposure after acute ingestion is likely too brief to result in substantial membrane incorporation. Thus, the targets are more likely rapid reactors such as transcriptional factors. NF-κB is a redox sensitive transcription factor that regulates the gene expression of many inflammatory proteins and has recently been shown to contribute to endothelial dysfunction in overweight and obese adults [31].

Interestingly, while the activation of NF-κB is considered a pro-inflammatory signal, we did not observe a subsequent increase in circulating inflammatory marker concentration following the high n-3FA meal (O3FA). Aljada et al [24] observed an increase in nuclear NF-κB binding in PBMC as well as expression of enzymes that activate this transcriptional factor within an hour of ingestion of a mixed meal. This was followed by a 28% increase in plasma CRP 3 h after the meal but no change in sICAM. The lack of association between upregulation of NF-κB by O3FA and inflammatory proteins in our study may be related to an insufficient duration of assessment or lack of measurement of other important transcriptional factors (e.g. AP1) or inflammatory factors (e.g. IL-6). For example, an increase in ICAM-1 following a butter-rich meal was not detected until 9 h postprandial [21], and another study showed postprandial effects at the mRNA level [32]. Evidence that other transcriptional factors may be affected by fatty acids was provided by a study demonstrating that the saturated fat-induced IL-6 release from cultured cells was independent of the NF-κB pathway [33].

Few clinical trials have examined the acute inflammatory and signaling responses to different fatty acids in humans. Similar to our study, Bellido et al [21] reported that a high fat meal containing polyunsaturated fats increased NF-κB. However, unlike our study; they noted the same effect 3 h after ingestion of a high saturated fat meal. It is possible that differences in fat sources played a role, as they used butter as the saturated fat source, while we used palm oil. Butter contains substantial amounts of medium chain fatty acids and half the proportion of palmitate as palm oil. This difference in palmitate content of the fats could help explain differences in results since *in vitro *studies have shown that lower levels of palmitic acid induce activation but higher levels can result in suppression of NF-κB [33].

The discrepancy between the view of n-3FA as anti-inflammatory [34,35] and our observed increase in NF-κB following acute n-3FA could be related to an adaptive effect that occurs with repeated exposure to n-3FA. It is possible that acute increases in NF-κB following n-3FA consumption are transient and occur when the individual is relatively naïve to n-3FA (low habitual dietary intake). Acute activation of NF-κB in this situation could serve to upregulate antioxidant status over time [36] and buffer sensitivity to inflammatory stimuli. This is similar to the effects of acute bouts of exercise which are shown by some studies to acutely increase inflammation and oxidative stress, but chronically reduce systemic inflammation [37]. Future research into the mechanisms by which the high n-3FA meal increased NF-κB acutely and whether this response changes over time with repeated ingestion is necessary to better understand the health effects of dietary n-3FA.

Chronic ingestion of n-3FA has been reported to increase indicators of oxidative stress in some studies [38,39] but not others [40,41]. Few studies have evaluated the effects of acute ingestion of n-3FA on oxidative stress but the presence of many double bonds susceptible to oxidation theoretically provides a rationale for increased acute oxidative stress. Hall et al [42] observed a 48% increase in plasma 8-epi 6 h following ingestion of high fat meal containing 5 g of n-3FA. Plasma concentration of this indicator of lipid peroxidation did not significantly increase following our high n-3FA meal but the average level tended to rise at 4 h postprandial. It is possible that our sampling period or our lower dose of n-3FA were insufficient to detect a change in this marker of oxidative stress.

ICAM-1 is constitutively expressed on the surface of endothelial cells and its release into the blood increases in response to inflammatory stress [43]. Our study supports the observed increase in ICAM-1 following acute saturated fat ingestion by lean individuals consuming a meal high in butter [21] and another report of lower postprandial ICAM-1 (AUC) for an olive oil meal compared to a higher saturated fat meal [44]. Chronic consumption may also influence postprandial response to these fats as a high monounsaturated fat diet for 12 weeks prior to an acute monounsaturated fat challenge had a favorable effect on postprandial ICAM-1 levels compared to 12 weeks of a higher saturated fat diet prior to a high saturated fat meal in metabolic syndrome patients [45]. Although the predictive role of ICAM-1 in CVD risk is unclear, it has been associated with future myocardial infarction risk [46], thus, the mechanism and implications of a postprandial increase in ICAM-1 deserve further study.

In regards to postprandial effects of high fat meals on triglyceride (TG) levels, our findings are contrary to Zampelas et al who showed that a meal high in fish oil reduced postprandial TG [47]. On the other hand, Jackson et al did not show differences in acute postprandial TG levels following an n-3FA rich meal in comparison to other fats [48]. Clearly, there is insufficient evidence in this area; and our results add to the little information available regarding the acute effects of n-3FA on postprandial TG. Interestingly, we also noted lower levels of postprandial TG following SFA compared to MFA. This is in line with Pacheco et al [44] who also noted lower TG over an 8 h period following a high palmitic sunflower oil meal compared to an olive oil meal.

One criticism of our study could be a relatively small number of subjects. However, a number of other studies reporting postprandial inflammatory response differences among different meals used a similar sample size [20,21,24], and since all subjects served as their own controls, variability between treatments was minimized. It is unlikely that testing more similar subjects would provide a different result for those factors that showed almost parallel responses following meals (e.g. VCAM-1, CRP). We did, however, calculate the number of subjects that would be necessary to achieve statistical difference between meal trials with 80% power for TNF-α and 8 epi. A total of 359 and 74 subjects would have been needed to allow determination that the trials caused differential responses (area under the curve) in TNF-α and 8 epi, respectively. This was not feasible and suggests that any difference in effect of the fats on these variables is very modest, if it exists at all, and that the variability in the measures is substantial. It is recommended that future studies including these measures attempt to reduce variability by choosing subjects with similar baseline inflammatory markers to attempt to address this. Our results may also only be applicable to overweight and obese individuals, although this is quite a large proportion of the population.

Overall, consumption of a high fat meal in our overweight subjects resulted in mixed effects on inflammatory markers, most of which were not robust. While some studies have shown that high fat meals lead to acute increases in some markers of inflammation and oxidative stress [19,49], others report inconsistent responses [6,50,51]. It is possible that a longer sampling period or measurement of gene expression and other gene products that may be more responsive to postprandial stresses was needed. For example, one lab reported significant increases in mRNA for TNF-α [52], transcriptional factors (AP-1, Egr-1), and matrix metalloproteinases [53] following acute glucose ingestion and another reported increased IL-6 mRNA following high fat meals [32]. In addition, studies reporting robust inflammatory responses to meals typically used subjects with insulin resistance [54], diabetes [19,20], or elevated baseline inflammation [55]. Our subjects may have been too healthy to trigger a major inflammatory state following the meals.

## Conclusions

While most of the inflammatory factors measured had modest or no change following the meal, ICAM-1 and NF-κB responded differently by meal type. These results suggest that type of fat in meals can differentially influence postprandial inflammation and endothelial activation. Additional work is needed in this area to determine the long term effects and/or whether adaptation occurs with repeated ingestion of these fats.

## List of abbreviations

BMI: body mass index; n-3FA: long chained omega fats (EPA or DHA); O3FA: high long chained omega fatty acids (EPA + DHA) meal; SFA: high saturated fat meal; MFA: high monounsaturated fat meal; PUFA: polyunsaturated fatty acids; CRP: C-reactive protein; TNF-α: tumor necrosis factor-α; ICAM-1: soluble intercellular adhesion molecule-1; VCAM-1: soluble vascular cell adhesion molecule-1; 8-epi: 8-epi-prostaglandin-F_2α_; NF-κB: nuclear factor - κB.

## Competing interests

The authors declare that they have no competing interests.

## Authors' contributions

Study conception and design (ATP, JWR, YWL); generation, collection, analysis and interpretation of the data (ATP, JWR, YWL), drafting and revision of manuscript (ATP, JWR), funding (JWR), approval of final manuscript (ATP, JWR, YWL).

## References

[B1] Dandona P Aljada A Chaudhuri A Mohanty P Garg R Metabolic syndrome: a comprehensive perspective based on interactions between obesity, diabetes, and inflammation Circulation 2005 111 1448 54 10.1161/01.CIR.0000158483.13093.9D 15781756

[B2] Basu A Devaraj S Jialal I Dietary factors that promote or retard inflammation Arterioscler Thromb Vasc Biol 2006 26 995 1001 10.1161/01.ATV.0000214295.86079.d1 16484595

[B3] Wing RR Phelan S Long-term weight loss maintenance Am J Clin Nutr 2005 82 222S 225 16002825 10.1093/ajcn/82.1.222S

[B4] Ceriello A Effects of macronutrient excess and composition on oxidative stress: relevance to diabetes and cardiovascular disease Curr Atheroscler Rep 2006 8 472 476 10.1007/s11883-006-0022-z 17045073

[B5] Neri S Calvagno S Mauceri B Misseri M Tsami A Vecchio C Mastrosimone G Di Pino A Maiorca D Judica A Romano G Rizzotto A Signorelli SS Effects of antioxidants on postprandial oxidative stress and endothelial dysfunction in subjects with impaired glucose tolerance and type 2 diabetes Eur J Nutr 2010 49 409 416 10.1007/s00394-010-0099-6 20213326

[B6] Patel C Ghanim H Ravishankar S Sia CL Viswanathan P Mohanty P Dandona P Prolonged reactive oxygen species generation and nuclear factor-kappaB activation after a high-fat, high-carbohydrate meal in the obese J Clin Endocrinol Metab 2007 92 4476 9 10.1210/jc.2007-0778 17785362

[B7] Kolovou G Anagnostopoulou K Daskalopoulou S Mikhailidis D Cokkinos D Clinical relevance of postprandial lipaemia Current Medicinal Chemistry 2005 12 1931 1945 10.2174/0929867054546609 16101498

[B8] O'Keefe JH Bell DSH Postprandial Hyperglycemia/Hyperlipidemia (Postprandial Dysmetabolism) Is a Cardiovascular Risk Factor The American Journal of Cardiology 2007 100 899 904 10.1016/j.amjcard.2007.03.107 17719342

[B9] Bansal S Buring JE Rifai N Mora S Sacks FM Ridker PM Fasting compared with nonfasting triglycerides and risk of cardiovascular events in women JAMA 2007 298 309 316 10.1001/jama.298.3.309 17635891

[B10] Kris-Etherton P Daniels SR Eckel RH Engler M Howard BV Krauss RM Lichtenstein AH Sacks F St. Jeor S Stampfer M Grundy SM Appel LJ Byers T Campos H Cooney G Denke MA Kennedy E Marckmann P Pearson TA Riccardi G Rudel LL Rudrum M Stein DT Tracy RP Ursin V Vogel RA Zock PL Bazzarre TL Clark J AHA Scientific Statement: Summary of the Scientific Conference on Dietary Fatty Acids and Cardiovascular Health: Conference Summary From the Nutrition Committee of the American Heart Association J Nutr 2001 131 1322 1326 11285345 10.1093/jn/131.4.1322

[B11] Robinson LE Buchholz AC Mazurak VC Inflammation, obesity, and fatty acid metabolism: influence of n-3 polyunsaturated fatty acids on factors contributing to metabolic syndrome Appl Physiol Nutr Metab 2007 32 1008 1024 10.1139/H07-087 18059573

[B12] Adkins Y Kelley DS Mechanisms underlying the cardioprotective effects of omega-3 polyunsaturated fatty acids J Nutr Biochem 2010 21 781 792 10.1016/j.jnutbio.2009.12.004 20382009

[B13] Tholstrup T Sandstrom B Bysted A Holmer G Effect of 6 dietary fatty acids on the postprandial lipid profile, plasma fatty acids, lipoprotein lipase, and cholesterol ester transfer activities in healthy young men Am J Clin Nutr 2001 73 198 208 11157314 10.1093/ajcn/73.2.198

[B14] Berry SE Tucker S Banerji R Jiang B Chowienczyk PJ Charles SM Sanders TA Impaired postprandial endothelial function depends on the type of fat consumed by healthy men J Nutr 2008 138 1910 1914 18806100 10.1093/jn/138.10.1910

[B15] Fahs CA Yan H Ranadive S Rossow LM Agiovlasitis S Wilund KR Fernhall B The effect of acute fish-oil supplementation on endothelial function and arterial stiffness following a high-fat meal Appl Physiol Nutr Metab 2010 35 294 302 10.1139/H10-020 20555373

[B16] Armah CK Jackson KG Doman I James L Cheghani F Minihane AM FIsh oil fatty acids improve postprandial vascular reactivity in healthy men Clin Sci 2008 114 679 686 10.1042/CS20070277 18052925

[B17] Burdge GC Calder PC Plasma cytokine response during the postprandial period: a potential causal process in vascular disease? Br J Nutr 2005 93 3 9 10.1079/BJN20041282 15705218

[B18] Ceriello A Quagliaro L Piconi L Assaloni R Da Ros R Maier A Esposito K Giugliano D Effect of postprandial hypertriglyceridemia and hyperglycemia on circulating adhesion molecules and oxidative stress generation and the possible role of simvastatin treatment Diabetes 2004 53 701 710 10.2337/diabetes.53.3.701 14988255

[B19] Nappo F Esposito K Cioffi M Giugliano G Molinari AM Paolisso G Marfella R Giugliano D Postprandial endothelial activation in healthy subjects and in type 2 diabetic patients: Role of fat and carbohydrate meals J Am Coll Cardiol 2002 39 1145 1150 10.1016/S0735-1097(02)01741-2 11923038

[B20] Carroll MF Schade DS Timing of antioxidant vitamin ingestion alters postprandial proatherogenic serum markers Circulation 2003 108 24 31 10.1161/01.CIR.0000074221.68903.77 12821556

[B21] Bellido C Lopez-Miranda J Blanco-Colio LM Perez-Martinez P Muriana FJ Martin-Ventura JL Marin C Gomez P Fuentes F Egido J Perez-Jimenez F Butter and walnuts, but not olive oil, elicit postprandial activation of nuclear transcription factor {kappa}B in peripheral blood mononuclear cells from healthy men Am J Clin Nutr 2004 80 1487 1491 15585759 10.1093/ajcn/80.6.1487

[B22] Andrews N Faller D A rapid micropreparation technique for extraction of DNA-binding proteins from limiting numbers of mammalian cells Nucl Acid Res 1991 19 2499 10.1093/nar/19.9.2499 PMC329467 2041787

[B23] Bierhaus A Wolf J Andrassy M Rohleder N Humpert PM Petrov D Ferstl R von Eynatten M Wendt T Rudofsky G Joswig M Morcos M Schwaninger M McEwen B Kirschbaum C Nawroth PP A mechanism converting psychosocial stress into mononuclear cell activation Proc Natl Acad Sci USA 2003 100 1920 1925 10.1073/pnas.0438019100 12578963 PMC149934

[B24] Aljada A Mohanty P Ghanim H Abdo T Tripathy D Chaudhuri A Dandona P Increase in intranuclear nuclear factor kappaB and decrease in inhibitor kappaB in mononuclear cells after a mixed meal: evidence for a proinflammatory effect Am J Clin Nutr 2004 79 682 690 15051615 10.1093/ajcn/79.4.682

[B25] Milne GL Sanchez SC Musiek ES Morrow JD Quantification of F2-isoprostanes as a biomarker of oxidative stress Nature Protocols 2007 2 221 226 10.1038/nprot.2006.375 17401357

[B26] Toborek M Lee YW Garrido R Kaiser S Hennig B Unsaturated fatty acids selectively induce an inflammatory environment in human endothelial cells Am J Clin Nutr 2002 75 119 125 11756069 10.1093/ajcn/75.1.119

[B27] Chen W Esselman WJ Jump DB Busik JV Anti-inflammatory effect of docosahexaenoic acid on cytokine-induced adhesion molecule expression in human retinal vascular endothelial cells Invest Ophthalmol Vis Sci 2005 46 4342 4347 10.1167/iovs.05-0601 16249517 PMC1378111

[B28] Jia Y Turek JJ Altered NF-[kappa]B gene expression and collagen formation induced by polyunsaturated fatty acids J Nutr Biochem 2005 16 500 506 10.1016/j.jnutbio.2005.01.016 16043032

[B29] Ross JA Eicosapentaenoic acid perturbs signalling via the NFkappaB transcriptional pathway in pancreatic tumour cells Int J Oncology 2003 23 1733 1738 14612948

[B30] Calder PC n-3 Polyunsaturated fatty acids, inflammation, and inflammatory diseases Am J Clin Nutr 2006 83 S1505 1519 10.1093/ajcn/83.6.1505S 16841861

[B31] Pierce GL Lesniewski LA Lawson BR Beske SD Seals DR Nuclear factor-{kappa}B activation contributes to vascular endothelial dysfunction via oxidative stress in overweight/obese middle-aged and older humans Circulation 2009 119 1284 1292 10.1161/CIRCULATIONAHA.108.804294 19237660 PMC2810548

[B32] Jimenez-Gomez Y Lopez-Miranda J Blanco-Colio LM Marin C Perez-Martinez P Ruano J Paniagua JA Rodriguez F Egido J Perez-Jimenez F Olive oil and walnut breakfasts reduce the postprandial inflammatory response in mononuclear cells compared with a butter breakfast in healthy men Atherosclerosis 2009 204 e70 6 10.1016/j.atherosclerosis.2008.09.011 18952211

[B33] Ajuwon KM Spurlock ME Palmitate Activates the NF-{kappa}B Transcription Factor and Induces IL-6 and TNF{alpha} Expression in 3T3-L1 Adipocytes J Nutr 2005 135 1841 1846 16046706 10.1093/jn/135.8.1841

[B34] El Seweidy MM El-Swefy SE Abdallah FR Hashem RM Dietary fatty acid unsaturation levels, lipoprotein oxidation and circulating chemokine in experimentally induced atherosclerotic rats J Pharm Pharmacol 2005 57 1467 1474 16259780 10.1211/jpp.57.11.0013

[B35] Carluccio MA Massaro M Bonfrate C Siculella L Maffia M Nicolardi G Distante A Storelli C De Caterina Raffaele Oleic Acid Inhibits Endothelial Activation: A Direct Vascular Antiatherogenic Mechanism of a Nutritional Component in the Mediterranean Diet Arterioscler Thromb Vasc Biol 1999 19 220 228 10.1161/01.ATV.19.2.220 9974401

[B36] Kesavulu MM Kameswararao B Apparao C Kumar EGTV Harinarayan CV Effect of omega-3 fatty acids on lipid peroxidation and antioxidant enzyme status in type 2 diabetic patients Diab Metab 2002 28 20 26 11938024

[B37] Bruunsgaard H Physical activity and modulation of systemic low-level inflammation J Leukoc Biol 2005 78 819 835 10.1189/jlb.0505247 16033812

[B38] Grundt H Nilsen D Mansoor M Increased lipid peroxidation during long-term intervention with high doses of n-3 fatty acids (PUFAs) following an acute myocardial infarction Eur J Clin Nutr 2003 57 793 800 10.1038/sj.ejcn.1601730 12792664

[B39] Pedersen H Petersen M Major-Pedersen A Jensen T Nielsen NS Lauridsen ST Marckmann P Influence of fish oil supplementation on in vivo and in vitro oxidation resistance of low-density lipoprotein in type 2 diabetes Eur J Clin Nutr 2003 57 713 720 10.1038/sj.ejcn.1601602 12771973

[B40] Higdon JV Liu J Du SH Morrow JD Ames BN Wander RC Supplementation of postmenopausal women with fish oil rich in eicosapentaenoic acid and docosahexaenoic acid is not associated with greater in vivo lipid peroxidation compared with oils rich in oleate and linoleate as assessed by plasma malondialdehyde and F(2)-isoprostanes Am J Clin Nutr 2000 72 714 722 10966889 10.1093/ajcn/72.3.714

[B41] Tholstrup T Hellgren LI Petersen M Basu S Straarup EM Schnohr P Sandstrom B A solid dietary fat containing fish oil redistributes lipoprotein subclasses without increasing oxidative stress in men J Nutr 2004 134 1051 1057 15113944 10.1093/jn/134.5.1051

[B42] Hall WL Sanders KA Sanders TA Chowienczyk PJ A high-fat meal enriched with eicosapentaenoic acid reduces postprandial arterial stiffness measured by digital volume pulse analysis in healthy men J Nutr 2008 138 287 91 18203893 10.1093/jn/138.2.287

[B43] van de Stolpe A van der Saag PT Intercellular adhesion molecule-1 J Mol Med 1996 74 13 33 10.1007/BF00202069 8834767

[B44] Pacheco YM Lopez S Bermudez B Abia R Villar J Muriana FJ A meal rich in oleic acid beneficially modulates postprandial sICAM-1 and sVCAM-1 in normotensive and hypertensive hypertriglyceridemic subjects J Nutr Biochem 2008 19 200 205 10.1016/j.jnutbio.2007.03.002 17651961

[B45] Perez-Martinez P Moreno-Conde M Cruz-Teno C Ruano J Fuentes F Delgado-Lista J Garcia-Rios A Marin C Gomez-Luna MJ Perez-Jimenez F Roche HM Lopez-Miranda J Dietary fat differentially influences regulatory endothelial function during the postprandial state in patients with metabolic syndrome: from the LIPGENE study Atherosclerosis 2010 209 533 538 10.1016/j.atherosclerosis.2009.09.023 19818442

[B46] Ridker PM Hennekens CH Roitman-Johnson B Stampfer MJ Allen J Plasma concentration of soluble intercellular adhesion molecule 1 and risks of future myocardial infarction in apparently healthy men The Lancet 1998 351 88 92 10.1016/S0140-6736(97)09032-6 9439492

[B47] Zampelas A Peel AS Gould BJ Wright J Williams CM Polyunsaturated fatty acids of the n-6 and n-3 series: effects on postprandial lipid and apo-lipoprotein levels in healthy men Eur J Clin Nutr 1994 48 842 848 7889892

[B48] Jackson KG Robertson MD Fielding BA Frayn KN Williams CM Olive oil increases the number of triacylglycerol-rich chylomicron particles compared with other oils: an effect retained when a second standard meal is fed Am J Clin Nutr 2002 76 942 949 12399264 10.1093/ajcn/76.5.942

[B49] Esposito K Nappo F Giugliano F Giugliano G Marfella R Giugliano D Effect of dietary antioxidants on postprandial endothelial dysfunction induced by a high-fat meal in healthy subjects Am J Clin Nutr 2003 77 139 143 12499333 10.1093/ajcn/77.1.139

[B50] Devaraj S Wang-Polagruto J Polagruto J Keen CL Jialal I High-fat, energy-dense, fast-food-style breakfast results in an increase in oxidative stress in metabolic syndrome Metabolism 2008 57 867 70 10.1016/j.metabol.2008.02.016 18502272 PMC2692901

[B51] Blackburn P Despres J Lamarche B Tremblay A Bergeron J Lemieux I Couillard C Postprandial variations of plasma inflammatory markers in abdominally obese men Obesity 2006 14 1747 1754 10.1038/oby.2006.201 17062804

[B52] Aljada A Friedman J Ghanim H Mohanty P Hofmeyer D Chaudhary A Dandona P Glucose ingestion induces an increase in intranuclear nuclear factor [kappa]B, a fall in cellular inhibitor [kappa]B, and an increase in tumor necrosis factor [alpha] messenger RNA by mononuclear cells in healthy human subjects Metabolism 2006 55 1177 1185 10.1016/j.metabol.2006.04.016 16919536

[B53] Aljada A Ghanim H Mohanty P Syed T Bandyopadhyay A Dandona P Glucose intake induces an increase in activator protein 1 and early growth response 1 binding activities, in the expression of tissue factor and matrix metalloproteinase in mononuclear cells, and in plasma tissue factor and matrix metalloproteinase concentrations Am J Clin Nutr 2004 80 51 57 15213027 10.1093/ajcn/80.1.51

[B54] Neri S Signorelli SS Torrisi B Pulvirenti D Mauceri B Abate G Ignaccolo L Bordonaro F Cilio D Calvagno S Leotta C Effects of antioxidant supplementation on postprandial oxidative stress and endothelial dysfunction: a single-blind, 15-day clinical trial in patients with untreated type 2 diabetes, subjects with impaired glucose tolerance, and healthy controls Clin Ther 2005 27 1764 73 10.1016/j.clinthera.2005.11.006 16368447

[B55] Browning LM Krebs JD Moore CS Mishra GD O'Connell MA Jebb SA The impact of long chain n-3 polyunsaturated fatty acid supplementation on inflammation, insulin sensitivity and CVD risk in a group of overweight women with an inflammatory phenotype Diabetes, Obesity and Metabolism 2007 9 70 80 10.1111/j.1463-1326.2006.00576.x 17199721

